# Multidimensional Neural Selectivity in the Primate Amygdala

**DOI:** 10.1523/ENEURO.0153-19.2019

**Published:** 2019-10-11

**Authors:** Philip T. Putnam, Katalin M. Gothard

**Affiliations:** 1 Graduate Interdisciplinary Program in Neuroscience, University of Arizona, Tucson, Arizona 85724; 2 Department of Physiology, College of Medicine, University of Arizona, Tucson, Arizona 85724

**Keywords:** attention, emotion, face, limbic, mixed selectivity, social

## Abstract

The amygdala contributes to multiple functions including attention allocation, sensory processing, decision-making, and the elaboration of emotional behaviors. The diversity of functions attributed to the amygdala is reflected in the response selectivity of its component neurons. Previous work claimed that subsets of neurons differentiate between broad categories of stimuli (e.g., objects vs faces, rewards vs punishment), while other subsets are narrowly specialized to respond to individual faces or facial features (e.g., eyes). Here we explored the extent to which the same neurons contribute to more than one neural subpopulation in a task that activated multiple functions of the amygdala. The subjects (*Macaca mulatta*) watched videos depicting conspecifics or inanimate objects, and learned by trial and error to choose the individuals or objects associated with the highest rewards. We found that the same neurons responded selectively to two or more of the following task events or stimulus features: (1) alerting, task-related stimuli (fixation icon, video start, and video end); (2) reward magnitude; (3) stimulus categories (social vs nonsocial); and (4) stimulus-unique features (faces, eyes). A disproportionate number of neurons showed selectivity for all of the examined stimulus features and task events. These results suggest that neurons that appear specialized and uniquely tuned to specific stimuli (e.g., face cells, eye cells) are likely to respond to multiple other types of stimuli or behavioral events, if/when these become behaviorally relevant in the context of a complex task. This multidimensional selectivity supports a flexible, context-dependent evaluation of inputs and subsequent decision making based on the activity of the same neural ensemble.

## Visual Abstract

**Figure:**
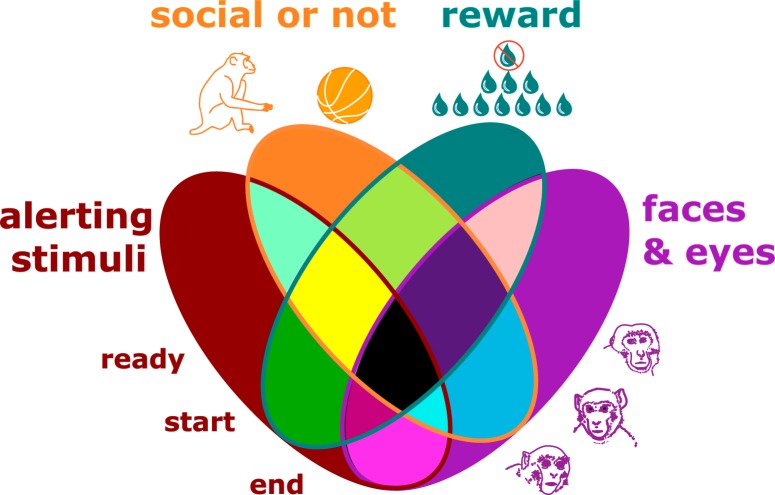

## Significance Statement

The primate amygdala contains neurons tuned to stimuli of high behavioral significance such as reward and punishment, faces, and eyes. It has been assumed that these specialized responses emerge from domain-specific cortical inputs that are evaluated for affective significance in the amygdala. Here we show that in the context of a task that requires the joint activation of multiple functions of the amygdala, neurons show multidimensional response properties (i.e., instead of specialization for relatively narrow domains of stimuli), they respond to multiple types of stimuli and multiple task events. This finding adds to growing experimental and theoretical evidence that the same neurons in the amygdala can serve, depending on the behavioral context, multiple functions.

## Introduction

The main role of the amygdala is to differentiate between rewarding or approach-inducing, and aversive or avoidance-inducing stimuli. Amygdala-dependent behaviors are based on multiple functions that emerge from the joint activity of subsets of neurons. These behaviors include but are not restricted to the following: defensive behaviors (for review, see LeDoux, 2003; Maren and Quirk, 2004); the coordination of autonomic responses (Pribram, 1967; Kapp et al., 1979; Amiez et al., 2003; Laine et al., 2009); attention and vigilance (for review, see Davis and Whalen, 2001); reward processing (for review, see Baxter and Murray, 2002; Morrison and Salzman, 2010); and social perception, including the differentiation of individuals (for review, see Gothard et al., 2007; Adolphs, 2010; Rutishauser et al., 2015). Each function underlying these behaviors is instantiated in the activity of neurons that appear specialized or tuned to a specific class of stimuli or events. It is unclear whether these neurons are exclusively active in response to a single type or multiple, possibly independent, types of stimuli or events.

In some experimental contexts, neurons in the amygdala segregate into stimulus-selective or task-related subpopulations (Beyeler et al., 2016; Kim et al., 2016), suggesting neuronal specializations within the amygdala. When animals choose between reward or punishment, approach or avoidance, or other mutually exclusive alternatives, neurons diverge along clear separation lines (Paton et al., 2006). These observations naturally led to the assumption that neurons in the amygdala are tuned to one of the alternatives. However, in the context of more complex tasks amygdala neurons show broader selectivity (Nishijo et al., 1988; Salzman and Fusi, 2010; Munuera et al., 2018) and multimodal responses (Morrow et al., 2019). The goal of this study was to examine neural responses in the primate amygdala during a task that quasi-simultaneously activated multiple, well characterized functions of the amygdala. This task required (1) attention to multiple, behaviorally relevant cues; (2) learning the value associated with different stimuli; (3) discrimination of social and nonsocial stimuli; and (4) discrimination between individuals. A putative role of oxytocin (OT) of modulating the expected social behaviors or the neural responses to social stimuli was also examined by exposing the subjects to vaporized oxytocin or saline before each experiment.

The task was designed to elicit within each trial a type of neural response that had been previously documented in less complex tasks. For example, alerting stimuli, such as the fixation icon or the onset/offset of a visual stimulus often elicit neural responses (Mosher et al., 2010), reflecting the role of the amygdala in attention and vigilance (Davis and Whalen, 2001). Neurons that respond to the fixation cue may also respond to subsequent stimuli that typically contain behaviorally relevant information (Mosher et al., 2010). Value-related neural responses have been amply documented in conditioning tasks, where distinct cues predict positive or negative valence (Paton et al., 2006; Livneh and Paz, 2012; Saez et al., 2017). Even the plan to obtain reward (Hernádi et al., 2015) and the propensity to consume or save rewards (Grabenhorst et al., 2012) elicit value-related neural responses in the primate amygdala. The idea that the same neurons may be part of multiple, even opposing, circuits was strongly suggested by the high degree of similarity between neurons in the rodent amygdala that predict appetitive and aversive outcomes (Shabel and Janak 2009). More recently, Kyriazi et al. (2018) reported that in the context of a risk–reward interaction task, neurons in the rat amygdala concurrently encode multiple stimulus and task features. Finally, a prominent role of the primate amygdala in social cognition has been evinced by neurons that differentiate between social and nonsocial stimuli (Gothard et al., 2007; Mosher et al., 2010; Minxha et al., 2017). Several authors proposed that the amygdala contains neuronal specializations for the representation of faces (Sanghera et al., 1979; Rutishauser et al., 2011), facial expressions (Gothard et al., 2007), eye contact (Mosher et al., 2014), and social vocalizations (Gadziola et al., 2016). These neurons, which appear specialized for the social domain, can also respond to nonsocial entities such as reward (Munuera et al., 2018). Here we combined four domains of selectivity to determine the extent to which subpopulations of neurons that were tuned to each domain belonged to overlapping or distinct groups. 

## Materials and Methods

### Subjects

All experimental and surgical procedures complied with guidelines of the National Institutes of Health for the use of primates in research, and were approved by the Institutional Animal Care and Use Committee. Behavioral and neural data were collected from monkeys M and H, two adult (8-year-old) male rhesus macaques (*Macaca mulatta*). Both animals were housed in double-sized cages, in the same room, with visual access to the other monkeys in the colony. They were implanted with custom-manufactured bilateral recording chambers (Thomas Recording) that allowed access, via bilateral craniotomies, to both amygdalae. For accurate eye tracking, the implants contained three small titanium posts for the attachment of a ring used for head fixation.

### Neurophysiological recordings

Single-unit activity was recorded bilaterally from both amygdalae using custom-made 16-channel linear V-probe electrodes (Plexon). The probes were advanced to their targets by custom-built NAN drives (NAN Instruments) or MEM drives (Thomas Recording) attached to the chamber. The wideband analog signal from the electrodes was digitalized via a head stage at 40 kHz (Plexon) and recorded using an OmniPlex neural data acquisition system (Plexon). The wideband data were then filtered on-line with a high-pass filter (600 Hz) to isolate single-unit activity. Spike sorting was performed off-line using the Offline Sorter (Plexon). We analyzed only units with a signal-to-noise ratio larger than 2:1 and with stability throughout the recording session (no abrupt changes in spike-wave form shape).

During recordings, the subject monkeys were seated in custom-built primate chairs with an LCD monitor spanning 38° × 40° of visual angle [degree of visual angle (DVA)] placed at 58 cm from their eyes. Eye movements were calibrated by fixating on a 9-point calibration grid within an error of ±1 DVA. Eye position was recorded using an infrared camera at 240 Hz (ISCAN) and sampled as an analog signal using an OmniPlex neural data acquisition system (Plexon).

### Behavioral task

**Figure 1.**
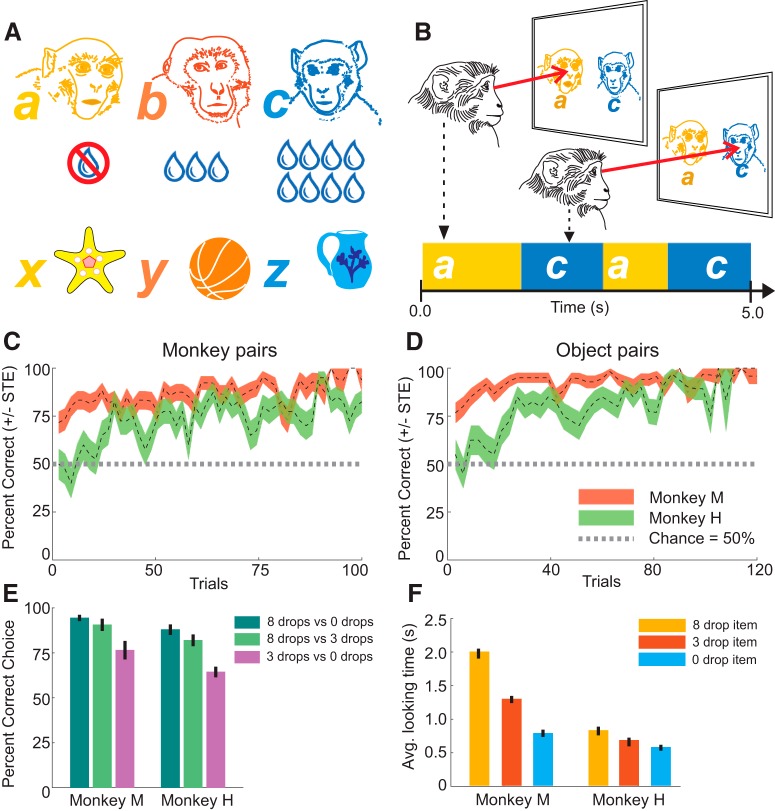
Behavioral task and performance. ***A***, During each recording session the subject encountered three unfamiliar monkeys and objects (a, b, and c). Each monkey and object was associated with a different amount of juice reward. The three items in each category were paired in the three combinations (ab, ac, and bc). Maximizing the reward was the incentive to discriminate between both monkeys and objects. ***B***, The periods of looking at each of the two videos displayed on the monitor was determined based on the viewer monkey’s eye movement. In this case, monkey a (zero drops) and monkey c (eight drops) are viewed in sequential looks, defined as a succession of fixations and saccades on the same video or same general gaze target (e.g., face or body). ***C***, ***D***, Correct performance was defined as selecting the higher value stimulus on each trial. Both subject animals performed above chance. ***E***, Choice accuracy was highest on trials contrasting stimuli associated with zero to eight drops of juice and the lowest for contrasting three to zero drops of juice. ***F***, Monkeys spent more time fixating on the higher valued stimuli.

Subjects were required to discriminate between videos of either freely behaving conspecifics (henceforth called stimulus monkeys) or videos of moving objects (Fig. 1). These videos were a proxy for social and nonsocial stimuli. We did not expect the movement of the inanimate objects to activate neurons that otherwise might respond to biological motion or other social behaviors. Both monkeys participated in 10 recording sessions. During each session the subjects encountered three previously unfamiliar stimulus monkeys and three unique objects (Fig. 1).

Each stimulus monkey or object was associated with a fixed amount of juice reward, as follows: eight drops, three drops, and zero drops of juice. On each trial, two of the three stimulus monkeys or objects were presented side by side in simultaneously displayed 5 s videos. Subjects were able to freely view both videos, before they were cued to select either the right or left video. Monkey M received a choice cue halfway through the video presentation (2.5 s) and then selected either the right or left video by touching and holding a right- or left-sided infrared button for 800 ms before the video ended to choose the corresponding stimulus monkey or object. Monkey H selected either the right or left video by fixating on a central fixation point following the video presentation and then by making a saccade to a blue square on the right or left side, corresponding to the stimulus shown on that side. This difference in task did not have a detectable effect on the outcome of the experiment. The neural correlates of the behaviors that were different between monkey M and monkey H were not analyzed. To prevent the monkeys from choosing an individual based on a specific video, each stimulus monkey and object was shown in 6 different video clips. These clips depicted different behaviors and were presented pairwise in a pseudorandomized order, where unique pairings of videos were presented only once. This prevented the subjects from choosing a stimulus monkey (or an object) based on low-level visual features, rather than extracting identity from multiple dynamic views of an individual as happens during natural social interactions. Monkeys were not otherwise rewarded or incentivized to view the stimuli. During the course of a recording session the subjects completed 25–30 blocks consisting of 10 monkey or 10 object trials. These blocks were intermixed.

The subjects initiated each trial by responding to a start cue. The chair of monkey M was fitted with three infrared buttons, and he initiated a trial by touching and holding the middle button for 500 ms. Monkey H initiated a trial by fixating (for 50 ms) on an icon presented at the center of the monitor. Following the initiation of each trial, the two videos were then immediately displayed simultaneously. The subjects were free to view either side of the monitor or to look away from the monitor while the videos were playing (Fig 1). Due to these differences in operant responses, the neural activity related to the choice behavior is not reported here. Monkey M received training with the stimuli the day before recording, and thus his behavior was above chance from the beginning of the recording session (Fig. 1*C*,*D*, orange line). As it became clear that the monkeys learned these association, rapidly the pretraining sessions were abandoned, and monkey H learned the stimulus–reward association during the recording session.

The task design included and additional manipulation that was expected to enhance neural individuation. Before each session, the monkeys received intranasal oxytocin or saline. With the exception of fewer neurons that responded to individual monkeys in one of the subjects, oxytocin did not alter the subjects’ behavior or the propensity of neurons to respond to a single or to multiple types of stimuli reflected by any of the measures used in the analyses reported here. In the absence of any effects that may have altered the outcome of the results presented here, we have pooled the neurons recorded in all sessions from both monkeys.

### Anatomic targeting and the reconstruction of recording sites

During recording, guide cannulae were inserted into a grid fitted into the chamber. The cannulae penetrated the dura and the cortex to a depth of 4–6 mm. V-probes were advanced through the guide cannulae to a depth calculated based on structural MR images (1 mm slice thickness). The boundaries of the main nuclei of the amygdala were outlined on each MRI slice. The anatomic reconstructions of electrode targets were based on a postsurgical MRIs that used columns of contrast positioned coaxially with the recording chambers, allowing us to calculate the *x*-*y*-*z* location of the each recording site in the amygdala relative to the chamber coordinates. The estimated *x*-*y*-*z* coordinates of recording sites were confirmed histologically (via small electrolytic lesions placed at known coordinates) in subject M. Subject H was involved in ongoing experiments. Single units recorded outside the amygdala were discarded from the analysis.

### Data analyses

All analyses were conducted using custom-designed programs in MATLAB R2018 (MathWorks). We determined whether the recorded neurons respond to or discriminate between one of the following four features: (1) task-related events; (2) categories of stimuli (monkeys vs objects); (3) individual stimulus monkeys or objects; and (4) faces or eyes. Instead of traditional spike train analyses, which can identify the selectivity of each neuron for these features separately, we used general linear models (GLMs) to capture the extent to which each neuron is tuned to all the features (the specifics of the GLM are described in more detail below). For example, GLMs give a quantitative measure of the extent to which neurons that are category selective for social stimuli also differentiate between individual items in that category such as the faces or the eyes of particular individuals. Response selectivity for each feature was computed from the segments of the spike train (details below) that followed the display of a particular stimulus or segment of time immediately following an eye movement that brought a particular stimulus or stimulus feature into central gaze. For example, selectivity for the alerting fixation icon was calculated based on the segment of the spike train that occurred immediately following the display of the trial start cue, whereas for assessing selectivity for individual faces we had to take into account the eye movements of the subjects. Instead of individual fixations and saccades (that might be shorter than the optimal window of analysis) we used “looks” [i.e., a consecutive sequence of saccades and fixation in the same area (e.g., face, body)].

### Classification of task-responsive neurons

Each cell was tested for responsiveness to the following four task events: (1) the start cue onset at the beginning of each trial (start cue); (2) the onset of stimulus presentation (video-on); (3) the end of stimulus presentation (video-off); and (4) the presentation of the choice cue (choice). To be classified as responsive to one of these events a cell was required to show a significant change in its firing rate during a postevent window (600 ms width, with an offset of +50 ms) when compared with a matched pretrial baseline period during the previous intertrial interval using a two-sample Kolmogorov– Smirnov (KS) test (*p* < 0.05). In addition to comparing the mean firing rate in these windows, we also required that the firing rate in at least one bin in the response window to be significantly different from the distribution of matched baseline bins (two-sample KS test, *p* < 0.05 corrected for multiple comparisons using a method described by Benjamini and Hochberg, 1995). For this analysis, trials were the independent observations. Neurons that were included in the analysis had to have a minimum of 75 trials.

### Classification of category, value, and identity selectivity

We tested the selectivity of neurons for (1) category, (2) associated value, and (3) identity by fitting different GLMs separately to each neuron. These models contained terms for category (social, nonsocial), value (eight, three, and zero drops of juice), or both category and value (with and without interaction terms). The response variable for all models was the spike count during a window spanning 50–350 ms after the onset of the look (recall that a look is a consecutive sequence of fixations and saccades on the same video). For comparison with a baseline model, a null model was created with the duration of the look as a random continuous variable, and this model was nested in all other subsequent models. Models containing a term for category (monkey or object as categorical variable) or value (eight, three, or zero drops of juice) were then compared with the baseline model to determine whether either term significantly improved the fit of the model. Comparisons of model fit were performed using a log likelihood ratio test (*p* < 0.05). GLMs were fit and compared using the MATLAB functions “fitglme” and “compare,” respectively. The link function (i.e. the relationship between the predictor variable and distribution function) was identity (Xβ = µ). If adding a term for either category or value (but not an interaction term) significantly improved the model fit compared with null, then the neuron was identified as being selective for category or value, respectively. Because, in this task, identity could be defined as a single category–value pairing, if the addition of an interaction term for category and value improved the fit compared with all other models, the neuron was considered selective for identity (because identity could was defined as a single category–value pairing). For this analysis, each look was an independent observation.

### Classification of face and eye selectivity

We tested the selectivity of neurons for fixations on the face or eyes by fitting different GLMs separately to each neuron. For this analysis only, those 50–350 ms segments of the spike train were used that corresponded to looks that landed on either the face/eyes or body of monkeys in videos. Here again, a null model was created with a duration of the look in milliseconds as a random continuous nuisance variable, and this model was nested in all other subsequent models. A model containing a term for fixation target (eyes/face or body as a variable was then compared with the baseline model to see whether either term significantly improved the fit of the model. Comparisons of model fit were performed as described above for task-related neurons. If adding a term for fixation target on the face or eyes significantly improved the fit of the model, then the cell was classified as eye/face selective.

To ascertain that the multidimensional neurons reported here are not a special category of cells in the amygdala, we determined whether these cells showed the same modulation in response duration, magnitude, and response polarity as described by Mosher et al. (2010). Specifically, we quantified response duration (phasic or tonic), response magnitude, and response polarity (significant increases or decreases of firing rates). Response duration <150 or >150 ms was classified as phasic or tonic, respectively. Response magnitude was calculated using the explained variance (sum of squares between groups by the sum of squares total) from a one-way ANOVA or the test statistic from a two-sample KS test, comparing either to baseline or across conditions as previously described. Response magnitude was *z*-scored across the entire population of cells to normalize against other values. The normalized average magnitude of selectivity was taken as the average of all response magnitudes for which that cell was responsive to or selective for.

### Effect of oxytocin on the examined parameters

Twenty minutes before the beginning of each recording session, subjects received an intranasal administration of either 50 IU oxytocin (Santa Cruz Biotechnology) in 2 ml of saline or 2 ml of saline vehicle (Pari) via a pediatric nebulizer. Each subject performed an equal number of sessions following OT (*n* = 5) or saline vehicle (*n* = 5) administration. We modeled a binomial distribution around the conditional probability that OT did not influence the frequency of the above classifications [Where P is the probability, *P*(OT-classification) = *P*(OT) * *P*(classification)] We then examined whether our observed frequency for each classification, under OT or saline, lay within the center 95% of this distribution (two-tailed test, α = 0.025). The only parameter that may have been significantly affected by oxytocin was the frequency of monkey identity-selective neurons (*p* = 0.0095), with significantly fewer monkey identity-selective cells observed following oxytocin inhalation in monkey H. This monkey also had additional experience with the stimuli (Fig. 1, difference in learning curves), and thus it is not clear that the observed effects were due solely to oxytocin. This effect was seen only on monkey H and, given that this result would only reduce the probability of finding multidimensional neurons, the data recorded under oxytocin and saline were combined for all other analyses, resulting in a more conservative approach.

For an additional examination of the potential effects of oxytocin on the multidimensional effects pursued in this study, we tested whether there was a significant relationship between the probability of a neuron recorded after oxytocin administration and selectivity for any of the four macro classifications of selectivity used to examine multidimensional selectivity. There was no significant relationship between oxytocin and the probability of neuron to respond to task events [*X* ^2^ (1, *N* = 308) = 0.92, *p* = 0.34] or to be category selective [*X* ^2^ (1, *N* = 308) = 0.76, *p* = 0.38]. Neurons that were identity selective, showed a significant relationship with oxytocin administration (*p* = 0.04). A significantly smaller proportion of monkey identity-selective cells was observed following oxytocin inhalation in monkey H [*X* ^2^ (1, *N* = 308) = 4.26], but not in monkey M [*X* ^2^ (1, *N* = 308) = 0.48, *p* = 0.49]. There was no significant relationship between oxytocin administration and the probability of a neuron to be selective to faces/eyes [*X* ^2^ (1, *N* = 308) = 0.004, *p* = 0.95].

## Results

Both subjects performed the discrimination task above chance (selecting the higher rewarded stimuli, bootstrap statistical test with *B* = 1000, *p* < 0.001) for both monkey stimuli (Fig. 1*C*) and object stimuli (Fig. 1*D*). Performance was dependent on the juice drop reward differential between the two stimuli presented; both subjects performed best on trials contrasting zero and eight juice drop stimuli, and performed the worst on trials contrasting zero and three juice drop stimuli (one-way ANOVA with *post hoc* multiple-comparisons test: monkey M, *p* = 0.0034, η^2^ = 0.237; monkey H, *p* = 0.000025, η^2^ = 0.544; Fig. 1*E*). Despite individual variation in looking time, both monkeys spent more time fixating on the higher valued stimuli (Fig. 1*F*; one-way ANOVA with *post hoc* multiple-comparisons test, monkey M, *p* = 0.0011, η^2^ = 0.311; monkey H, *p* = 0.0072, η^2^ = 0.374).

### Amygdala neurons were tuned to discrete features of the identity discrimination task

We analyzed the activity of 308 well isolated neurons from the left and right amygdala of two monkeys (monkey H = 202; monkey M = 106). The selectivity of each neuron in this population was tested for four task- or stimulus-related features: (1) task-related events, (2) categories of stimuli (monkeys vs objects), (3) individual stimulus monkeys or objects, and (4) faces or eyes. Note that there is an overlap between reward and identity because each monkey was associated with a single reward value (eight drops, three drops, and zero drops). As there are also objects uniquely associated with the same three reward levels, we could extract reward value independently of category (monkey and object). First, we report selectivity for any of these categories (including reward value), and then we report the probability of each neuron responding to more than one category (multidimensional selectivity).

### Selectivity for each category

**Figure 2.**
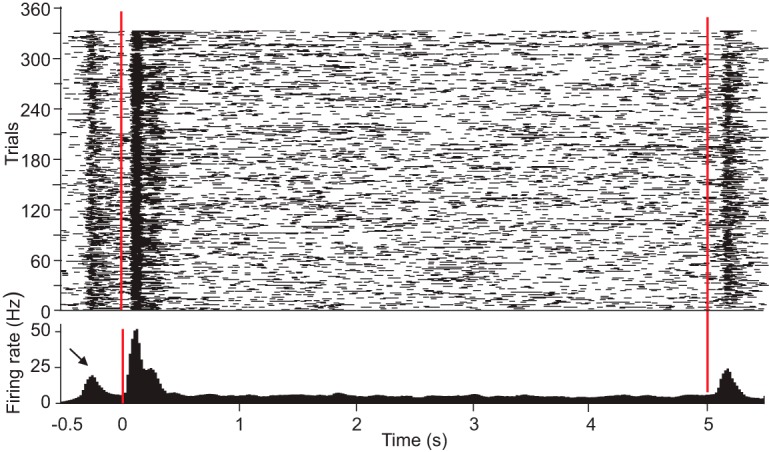
Raster plot and perievent time histogram for a neuron that responded to the start cue and also the onset and offset of the video, indicated by the vertical red lines. The black arrow indicates the increased firing rate elicited by the start cue that preceded the video onset. Due to a variable (±100 ms) delay between the start cue and video onset, the fast rise in firing rate elicited by the start cue is not as clearly aligned in time as the onset and offset of the video.

First, we identified task-responsive neurons that responded to (1) the presentation of the start cue, (2) the start of the video presentation, (3) the end of the video presentation, or (4) the presentation of the choice cue. The majority of the 308 recorded neurons (57.79%) significantly changed their firing rate to one or more of these task events (29 neurons in monkey M and 149 neurons in monkey H; recall that the final analyses included 106 neurons from monkey M and 202 neurons from monkey H). The task-responsive neuron shown in Figure 2 responded to the start cue, video onset, and video end.

**Figure 3.**
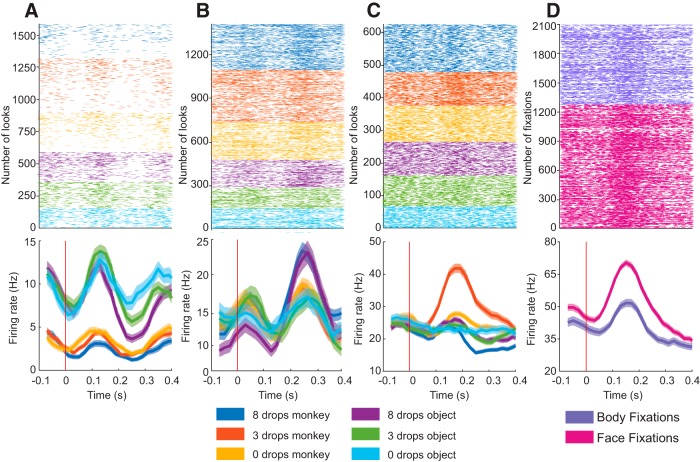
Raster plots and perievent time histogram for neurons tuned to different stimulus features. ***A***, Category-selective neuron that responded with higher firing rates to objects compared with monkeys (firing rates before the start of each look are offset because during the previous look the monkey was attending to the same category; on each trial, only objects or monkeys but not both were shown on the monitor). ***B***, Value-selective neuron that increased its firing rate in response to the monkey and the object that were associated with the highest reward. ***C***, Identity-selective neuron that increases its firing rate when the subject was looking at a specific monkey. ***D***, Face-selective neuron. Neural activity is aligned to either fixations on the face or eyes (shown in pink), or fixations on the body (shown in purple).

Second, we identified neurons that differentiated between broad categories of stimuli (monkeys and objects; Fig. 3*A*). Given that two videos were displayed on the monitor at the same time, we established selectivity by calculating the firing rate during each look (sequence of fixations and saccades on the same video). As shown previously (Gothard et al., 2007), nearly half of the recorded population of neurons (45.78%) were category selective (40 neurons in monkey M and 101 neurons in monkey H). The category-selective neuron shown in Figure 3*A* responded with higher firing rates to objects compared with monkeys (Fig. 3*C*, monkey-selective neuron). A smaller proportion of neurons was value selective (12.33%, 8 neurons from monkey M and 30 neurons from monkey H). The example value-selective neuron shown in Figure 3*B* had a higher firing rate for the high reward stimulus, either monkey or object. This type of selectivity was rare. More frequently, we found that neurons that responded to a combination of category and reward value (e.g., three-drop monkey and eight-drop objects). We identified the neurons that were both category selective and value selective (21.43%, 17 neurons from M and 49 from monkey H). Because each monkey or object was associated with a reward value, neurons that were selective for both category and value were also responding to the unique identity of the stimulus. For these neurons, the inclusion of an interaction term between category (monkeys or object) and value (eight, three, and zero drops of juice) into the GLM significantly improved the fit compared with other models, and so they were classified as identity-selective neurons. The identity-selective neuron shown in Figure 3*C* responded selectively to the monkey associated with three drops of juice.

Finally, we identified neurons that responded to fixation on the faces or eyes of monkeys. Fixations that landed on the eyes or face were compared with fixations that landed on the neuron was classified as face/eye-selective (20.78%, 5 neurons from monkey M and 42 neurons from monkey H). An example face/eye-selective neuron is shown in Figure 3*D*.

### Multidimensional selectivity

**Figure 4.**
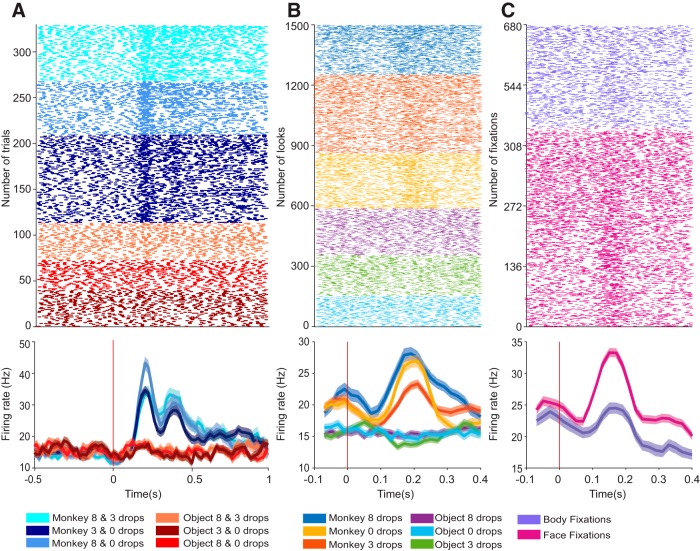
Example of a multidimensional neuron, tuned to multiple task and stimulus features. ***A***, Raster plots and perievent time histograms aligned to video onset and grouped by stimulus pairs (monkey pairs shown in shades of blue, object pairs shown in shades of red). Note that this neuron increased its firing rate to the presentation of monkey stimuli, demonstrating both a video onset response and category selectivity. ***B***, The same neuron also differentiated between the three monkeys. Here the neural activity is aligned to the onset of each look. ***C***, The same neuron was selective for fixations on faces, as shown by the neural activity aligned to the onset of fixations on the face or eyes of monkeys (shown in pink) compared with fixations on the body (shown in purple).

A large proportion of neurons (255 of 308 = 82.79%) showed selectivity for at least one of the events/stimulus features tested (71 neurons from monkey M and 184 neurons from monkey H). The majority of these neurons showed multidimensional responses (i.e., they were tuned to more than one task event or stimulus feature; 170 of 255 = 66.67%). Specifically, these 170 neurons met the criteria for more than one of the four possible classifications: (1) task, (2) category, (3) identity, and (4) face/eye selective. For example, neurons that signal the start cue may also be category selective and tuned to a particular item within a category. A multidimensional neuron is shown in Figure 4. This neuron responded to task events, was selective for monkeys, discriminated between identities (monkey–reward combinations), and preferred faces over bodies.

**Figure 5.**
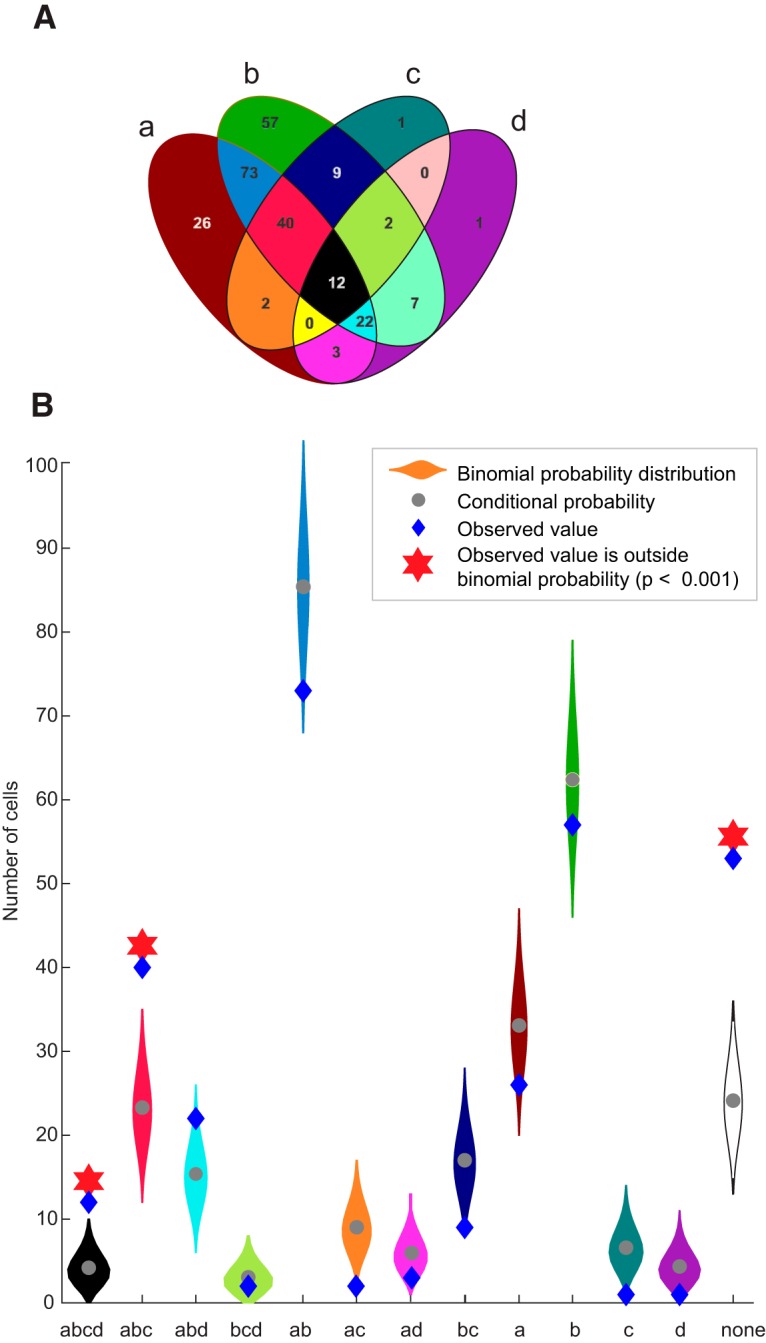
Distribution of selectivity combinations across the population of recorded neurons. ***A***, The nonproportional Venn diagram illustrates the four types of selectivity as four ellipses, where the intersectional areas of each ellipse correspond to neurons that show selectivity for the overlapping features. Neurons that are responsive to task events (start cue presentation, video onset or end, and presentation of the choice cue) populate the “a” ellipse. Neurons that were category selective or value selective populate the “b” ellipse. Identity-selective neurons populate the “c” ellipse. Finally, the “d” ellipse contains the neurons that were face/eye selective. The number of cells and the percentage of each combination of selectivity in the total population are marked inside each intersectional area. ***B***, A comparison between the theoretical and observed number of neurons in each type of selectivity (area shaded in each color as in ***A***). The theoretical distribution for each selectivity type was generated from a binomial probability distribution centered around the conditional probability calculated by assuming that the probability of a cell to respond selectively for any given level was independent of the selectivity for any other levels. This distribution is shown in the shaded areas (violin plots). The observed number of neurons in each combination of selectivity is indicated by the blue diamonds. A red star indicates when the observed number was outside 99.9% (corrected for two-tailed test and multiple comparisons) of the theoretical distribution.

We determined whether all or only certain mixtures of selectivity were found in the population of 170 multidimensional neurons. Given the four criteria for response selectivity, each neuron could fall into 1 of 16 possible combinations of selectivity. These include neurons that exhibited selectivity to all four classifications, neurons that were not selective to any of the four classifications, and all other possible combinations. The relative frequency of all permutations is illustrated in Figure 5*A* as a nonproportional Venn diagram. The number of cells we found in each selectivity combination is indicated by the blue diamonds in Figure 5*B*. Twelve neurons displayed selectivity to all four levels, and 53 neurons did not respond to any task event or stimulus parameter. Notably, there were only three combinations abd (fixspot + category + face), acd (fixspot + item + face) and bd (category + face) of selectivity levels for which we did not observe a representative neuron. This is explained by the low proportion of face-responsive neurons in our population.

To test whether the observed frequencies of occurrence for each combination of selectivity were different from what would be expected by chance (if the four classes of selectivity were completely independent of each other), we implemented a Monte Carlo simulation. A theoretical probability distribution (*n* = 10,000) was generated assuming that the chance of a neuron to meet the criteria for any of the four classes of selectivity was independent if it was included in the other classes. Importantly, for this simulation we retained the observed frequency of each class of selectivity in our sampled population. Then, the observed frequency of each combination of selectivity was compared with the theoretical distribution. A nonrandom occurrence was indicated by a frequency that lay significantly above or below the theoretical distribution (α = 0.025, two-tailed *t* test). Interestingly, the observed frequency of neurons that met the criteria for all four classes of selectivity was above the theoretical distribution (*p* < 0.00001). Likewise, the proportion of neurons that did not respond to any task event or stimulus feature, and the proportion of neurons with a particular combination of three classes of selectivity (abc = task + category + identity) was also significantly higher than chance. These findings may indicate that the propensity for multidimensional responses is a defining, and not spurious, feature of the neurons in the primate amygdala.

### Mixed selectivity

To evaluate the prevalence neurons that showed mixed selectivity, we performed a per-neuron ANOVA for each previously fit GLM containing fixed-effects terms for category, value, and a random effect of look length. After evaluating all 308 neurons considered in this study, we identified 11 neurons (11 of 308 = 3.57%) that exhibited mixed responses [as defined by significant (*p* < 0.05) interaction terms between category and value, but nonsignificant main terms of category and value]. In contrast, we identified 51 separate neurons where the ANOVA revealed both a significant (*p* < 0.05) fixed effect of category and/or value and a significant (*p* < 0.05) interaction term between category and value, suggesting that the prevalence of mixed-response neurons is relatively low compared to feature-encoding neurons.

Neurons that showed mixed or multidimensional selectivity were distributed across all major nuclei of the amygdala. To assess the anatomic distribution of multidimensional neurons, we reconstructed the nuclear origin of the recorded neurons by aligning the *x*-*y*-*z* coordinates of the recording V-probes to high-contrast fiducial markers on postoperative high-resolution MR images that allowed us to estimate the boundaries of the component nuclei.

The proportion of neurons that responded to more than one stimulus feature by nucleus was as follows: lateral nucleus = 11 neurons (64.7% of all lateral nucleus neurons); basal nucleus = 66 (50% of all basal nucleus neurons); accessory basal nucleus = 41 neurons (47.7% of all accessory basal nucleus neurons); central nucleus = 32 neurons (53.3% of all central nucleus neurons); and medial nucleus = 2 neurons (15.4% of all medial nucleus neurons). We found that the multidimensional neurons were equally likely in all the nuclei (one-way ANOVA, *p* = 0.12, η^2^ = 0.023). The electrophysiological characteristics of amygdala neurons were not linked to their multidimensional selectivity. Neurons were equally likely to respond with a decrease or increase in firing rate to different task parameters or stimuli (one-way ANOVA, *p* = 0.11, η^2^ = 0.028).

## Discussion

Here we report that the same neurons in the primate amygdala track multiple task events and multiple stimulus dimensions in the context of a reward-motivated identity discrimination task. The behavioral task combined multiple known functions of the amygdala that elicit predictable response types. Specifically, we activated within a trial neurons that respond to (1) alerting stimuli (Mosher et al., 2010), (2) broad categories of social versus nonsocial stimuli (Gothard et al., 2007), (3) faces (Sanghera et al., 1979; Minxha et al., 2017) and/or eye contact (Mosher et al., 2014), and (4) stimulus–reward associations (Paton et al., 2006). Tasks that pursued separately each of the these functions led to the conclusion that the amygdala contained “specialized” subpopulation of neurons that were tuned to distinct stimuli or stimulus features, such as faces, eyes, or the value associated with objects and events. Here we show that when subjects were required to keep track of multiple stimulus dimensions, neurons in the amygdala no longer separate in nonoverlapping, specialized subpopulations. On the contrary, the same neurons are recruited into different subpopulations, each population responding selectively to a different task or stimulus variable. Indeed, the same neurons encoded social and nonsocial dimensions of the stimuli even if they were not associated with the same reward value. For example, a neuron might respond selectively to the monkey associated with three drops of juice and also to the object associated with eight drops of juice. As Figure 5 shows, in a relatively small sample of 308 neurons of the 16 possible combinations of selectivity, we have found representative neurons for 13 combinations. Moreover, the number of neurons that showed selectivity for all four domains of selectivity examined here were above what would be expected by chance, suggesting that the observed multidimensional selectivity is not a mere accident resulting from the convergence of different inputs in the amygdala. A similar conclusion emerged recently from a study by Morrow et al. (2019), who tested the likelihood of neurons recorded from the monkey amygdala to respond to visual, tactile, and auditory stimuli. They found that the majority of these neurons are multimodal and have a higher probability of responding to stimuli of multiple sensory modalities than what would be expected by chance (Morrow et al., 2019). These multimodal and multidimensional responses are predicted by mathematical principles formulated in a recent theory of nonrandom combinatorial connectivity in cell assemblies (Li et al., 2016).

The idea that neurons that contribute to complex behaviors show selectivity for multiple task variables or stimulus parameters was proposed decades ago based on the responses properties of neurons in the hidden layer of artificial neural networks. Zipser and Andersen (1988) showed that the hidden layer of an artificial neural network (which received input from neurons reporting retinal position and eye position) contained neurons that responded to various combinations of the two inputs (gain fields). The response properties of these virtual neurons mapped onto the properties of neurons recorded from area 7a of posterior parietal cortex, an area involved in spatial perception. Moreover, neurons with mixed selectivity (i.e., nonlinear combinations of response properties) were reported in the prefrontal cortex of monkeys performing tasks that required alternating cognitive–behavioral strategies (Mante et al., 2013; Rigotti et al., 2013; Fusi et al., 2016). Elegant computational analyses showed that neurons with mixed selectivity are the signature of high-dimensional representations (Rigotti et al., 2013). High-dimensional representations are necessary for flexible, context-dependent behavioral options, typically present in high-level association areas such as the prefrontal cortex (Mante et al., 2013; Rigotti et al., 2013; Fusi et al., 2016) and the posterior parietal cortex (Raposo et al., 2014; Zhang et al., 2017). It appears that the monkey amygdala also contains a small number of neurons that meet the criteria for mixed selectivity matching similar reports from the human amygdala (Rutishauser et al., 2015; Farault et al., 2018). The number of cells that show mixed selectivity in our task is insufficient to directly compare the dimensionality of neural representations in the amygdala to what was shown for the neurons in the prefrontal cortex (Rigotti et al., 2013). However, the rich connectivity of the amygdala to the prefrontal cortex and its contribution to the majority of the behaviors attributed to the prefrontal cortex suggest that future studies will find not only multidimensional selectivity but also mixed selectivity, in the strict terms used by Rigotti et al. (2013). Indeed, the burden of coordinating complex behaviors is often carried by the amygdala in conjunction with distinct areas of the prefrontal cortex, and the coactivity pattern among different subregions highlights the differences in the division of labor among these areas (Belova et al., 2008; Morrison and Salzman, 2010; Saez et al., 2017; Pryluk et al., 2019).

Multidimensional representations are present in all brain areas that receive and process diverse inputs and generate context-dependent and state-dependent outputs (Wallis et al., 2001; Padoa-Schioppa and Assad, 2006; Shenoy et al., 2013). High-dimensional and often abstract representations at the population level translate at the single-neuron level into complex response properties. For example, the majority of face-responsive neurons in the monkey amygdala respond to unique combinations of face identity and facial expressions (Gothard et al., 2007). Taken separately, the identity or the emotional expression of a social partner may not be as informative for choosing a response strategy as the combination of identity and expression because the same emotional expression emitted by different social partners may require different actions in response. Social behavior depends on the functional integrity of the amygdala as it often requires the discrimination and the use of subtly distinct social signals (e.g., facial signals) that are expected to be high dimensional (Rutishauser et al., 2015). Multidimensional neurons were found in all sampled nuclei of the monkey amygdala, suggesting that multidimensional processing in the amygdala does not result from hierarchical convergence of specific anatomic pathways that target only a subset of nuclei (e.g., value signals from the prefrontal cortex target the basal and accessory basal but not the lateral, central, and medial nuclei; Ghashghaei et al., 2007). Neurons with multidimensional selectivity were also reported in the rodent amygdala (Grunfeld and Likhtik, 2018; Kyriazi et al., 2018) and were also distributed throughout the nuclei. These neurons may be key to explain the large and diverse array of behaviors in which the amygdala plays a significant role (Kennedy and Adolphs, 2012). Neural responses that mix two stimulus dimensions, such as social + reward (Munuera et al., 2018) or appetitive + aversive (Shabel and Janak 2009) have been shown both in monkeys and mice. Based on these findings, it is possible to argue that multidimensional responses convey to neural networks a level of degeneracy (Tononi et al., 1999) that is required for the creation of latent evolving variables during learning and flexible task switching (Cropper et al., 2016). Here we show that attention, categorization, reward magnitude, individuation, and face/eye selectivity could be processed by the same neurons. It appears, therefore, that narrow neural specializations, exemplified by “face cells” and “eye cells” are not the rule but the exception for neurons in the amygdala, and may be the result of not exposing these neurons to a sufficiently diverse set of stimuli and/or behavioral demands. In this study, only 15.3% of neurons responded to faces and eyes, and only 12.3% were value selective; it may be that the neurons in the amygdala appear “unresponsive” in tasks that are too simple and low dimensional to activate the neurons. Indeed, the current study and the recently published results of multimodal responses (Morrow et al., 2019) show that neurons that encode specific types of information are a minority (only 15.3% and 12.3% of face cells and reward cells, respectively) compared with multidimensional neurons, but this becomes obvious only when animals perform complex tasks or are placed in naturalistic behavioral contexts (Gothard et al., 2018).

A broader interpretation of the results presented here is limited by several factors. We report only 308 neurons recorded from two subjects. Despite these relatively small numbers, neurons exhibiting multidimensional selectivity were dominant in the population of cells recorded from both subjects. Although we consider these neurons as part of neural ensembles whose activity rises and falls simultaneously as the stimuli, the task, and the behavior of the animal enfolds, the number of neurons we recorded simultaneously with the two 16-channel V-probes are insufficient to address quantitatively the coactivity across subpopulations of cells. A technological advancement, which would increase the number of simultaneously monitored neurons by an order of magnitude, would be required to address issues of recruitment and derecruitment of neurons into neural ensembles. A further limitation is that the operant choice behavior was different for the two monkeys, and we were not able to include the dimensionality analyses of the neural responses to choice behaviors. Finally, the null effect of oxytocin administration adds to the growing evidence that the behavioral and neural effects of intranasal oxytocin in primates has not been unequivocally established (for review, see Putnam et al., 2018). Indeed, in humans and nonhuman primates, the large genetic and behavioral variation among individuals hinders the emergence of reliably replicable responses to oxytocin. Despite these limitations, the demonstration of multidimensional selectivity in the primate amygdala provides further evidence that the amygdala utilizes a common neural framework to process distinct task demands and stimulus parameters.

The four types of response selectivity examined in this study mark different levels on a continuum spanning from the most general to the most specific level of selectivity, and these levels were reflected in the proportion of neurons that responded to each level. We ranked responses to alerting stimuli such as task events as the most general level, as these responses were present in all trials (e.g., responses to the fixation icon, and to video-on and video-off events that required attention and engagement with the task, regardless of the stimuli). Responses to alerting stimuli reflect the role of the amygdala in coordinating general attention and vigilance, and were the most frequently observed responses in the population. Less frequent were the category-selective responses (differentiating monkeys from objects). These responses were more specific than responses to alerting stimuli, but were more general than the selectivity of individuals. Selectivity for individuals (that did not always require looking at the eyes) ranked in frequency below category selectivity but above face and eye selectivity, which represented the highest level of specificity. Note that multidimensional responses do not automatically imply inclusion in a more general/less specific level of selectivity (nested selectivity); for example, we found three neurons that responded selectively to faces and eyes + task events, but did not differentiate social and nonsocial stimuli or individuals. The inverse relationship between the specificity and frequency of a particular type of response (the most specific being the least frequent) justifies a shift of emphasis from neurons of pure selectivity (face cells) to neurons of mixed or multidimensional selectivity, especially in brain areas such as the prefrontal and parietal cortex that coordinate multiple cognitive functions (Rigotti et al., 2013; Zhang et al., 2017). Ultimately, it is likely that behaviorally meaningful brain states do not emerge from a higher number/proportion of neurons with highly selective response properties but from temporal interactions across large population of multidimensional neurons.

## Synthesis

Reviewing Editor: Mark Baxter, Mount Sinai School of Medicine Decisions are customarily a result of the Reviewing Editor and the peer reviewers coming together and discussing their recommendations until a consensus is reached. When revisions are invited, a fact-based synthesis statement explaining their decision and outlining what is needed to prepare a revision will be listed below.

The manuscript was accepted after editorial review.
